# Arterial Switch Operation for Transposition of the Great Arteries: Can We Do it Better?∗

**DOI:** 10.1016/j.jacadv.2023.100771

**Published:** 2023-12-22

**Authors:** Annette Schophuus Jensen

**Affiliations:** Rigshospitalet, Copenhagen University Hospital, Copenhagen, Denmark

**Keywords:** arterial switch operation (ASO), mortality, re-intervention, Taussig-Bing double outlet right ventricle (TB-DORV), transposition of the great arteries (TGA)

**Figure:**
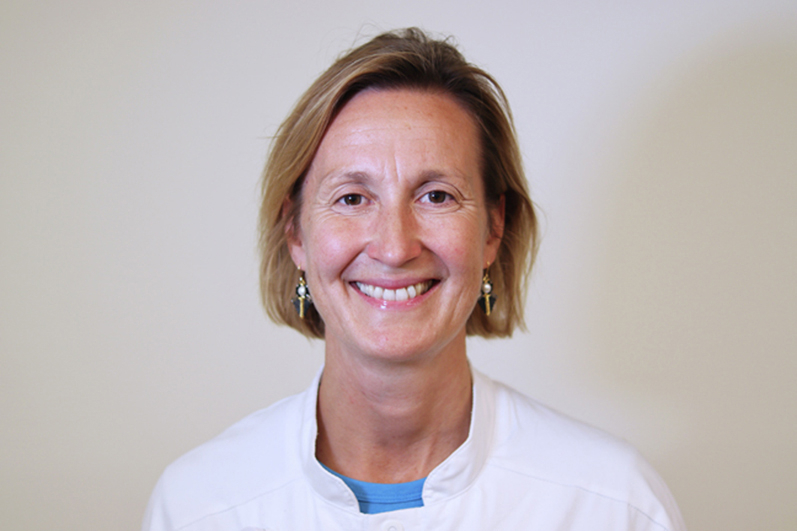

The natural history of transposition of the great arteries (TGA) is dismal, but several advancements in the treatment have improved the survival rate. Palliation with surgical atrial septostomy was described in 1950 by Blalock and Hanlon and modified to a catheter-based intervention in 1966 by Rashkind. The atrial septostomy allows neonates to survive until establishing a serial circulation, which initially was performed by atrial switch operation as introduced by Senning in 1957 and modified by Mustard in 1964. However, atrial switch introduces problems with the right ventricle and tricuspid valve being in the subaortic position, which may lead to heart failure, tricuspid regurgitation, and sudden cardiac death. Furthermore, there are several inherent problems with the extensive surgery in the atria often resulting in sinus node dysfunction, atrioventricular block, and atrial tachycardias. As early as 1975, Jantene reported his results for the arterial switch operation (ASO), and although the initial perioperative mortality was high, the survival improved during the following decades. Furthermore, since ASO is restoring a circulation which anatomically almost mirrors the normal heart, better long-term outcomes are expected as compared to after the atrial switch operation. However, several issues may impact the fate of patients after ASO:

• Right ventricular outflow tract (RVOT) obstruction at the arterial anastomosis as well as after pulmonary artery banding (PAB).
• Left ventricular outflow tract (LVOT) stenosis at the arterial anastomosis, or regurgitation of the anatomically pulmonary valve now serving as a neo-aortic valve.
• Coronary artery kinking at the site of reinsertion to the neo-aortic root, which was the main reason for early mortality when ASO was introduced but also can cause sudden cardiac death later in life.
• Atrial tachyarrhythmia caused by closure of the atrial septal defect after Rashkind or by the incision in the atrial wall during index surgical repair. Furthermore, atrioventricular block may occur due to, for example, closure of ventricular septal defect.

In this issue of *JACC: Advances*, Ingele et al^1^ used a nationwide prospective registry in the Netherlands to identify 1,061 patients with TGA who survived the first 30 days after ASO. The patients were divided into 3 subgroups: TGA with intact ventricular septum (TGA-IVS), TGA with ventricular septal defect, and Taussig-Bing double outlet right ventricle (TB-DORV) accounting for 66%, 29%, and 5%, respectively, of the study population. Palliation with PAB or Rashkind was performed in 4% and 31%, respectively.

It is important to notice that the registry had several important limitations, such as that only 75% to 80% of eligible patients were included, the median age at inclusion was 10.7 years (IQR: 2.0-18.2 years), and the median follow-up was 8.0 years (IQR: 5.4-8.8 years). Consequently, event rates may be underestimated and long-term outcomes should be interpreted with caution, particularly since only patients from the early ASO learning curve are represented at, for example, 35 years of age.

Keeping this in mind, long-term outcomes are promising with a 35-year survival rate of 93%, although patients with TB-DORV had only 60% survival. This is significantly better than what has been reported after atrial switch operation, where in particular the postoperative mortality was very high.^2^ Engele et al only provide limited granularity about causes of mortality after ASO. However, these also seem to be different from atrial switch operation where the most common causes were sudden cardiac death followed by heart failure/heart transplantation.^3^

Although mortality was low after ASO, the need for re-intervention was high in the registry from the Netherlands. Thus, 240 procedures were performed in 144 (13.6%) patients with TB-DORV, and previously, PAB was associated with the need for earlier and more frequent re-intervention. The most common cause of re-intervention was related to the RVOT, where 77 catheter-based and 50 surgical procedures were performed with an estimated cumulative incidence of 36% at the age of 35 years.

Re-intervention on LVOT was performed in 37 cases, for example, neo-aortic root and neo-aortic valve with Bentall procedure and neo-aortic valve replacement as the most common procedures. At 35 years of age, the estimated cumulative incidence of LVOT intervention was 16%.

Aortic arch re-intervention was done with a cumulative incidence of 9% of the patients at age 35, with equal distribution between surgery and transcatheter intervention with balloon dilatation or stenting.

Although ASO can currently be performed at a low surgical risk, a higher mortality was seen when the therapy gradually replaced the atrial switch. This was mainly due to the translocation of the coronary arteries and concerns regarding later issues with the coronary arteries have been raised. Therefore, it is to some degree re-ensuring that the cumulative incidence of coronary artery bypass grafting and percutaneous coronary intervention was only 3% at age 35 years.

Catheter-based radio-frequency ablation was performed in 1.8% of the patients due to supraventricular (n = 11) or ventricular (n = 8) tachyarrhythmia. In addition, pacemaker and implantable cardioverter defibrillator were used in 0.5% and 0.8%, respectively. The estimated cumulative incidence of any electrophysiological intervention at age 35 years was 11%.

In addition to the abovementioned intervention, 1.6% of the patients had at least 1 clinical event including infectious endocarditis (n = 8), heart failure (n = 8), myocardial infarction (n = 2), and cardiac arrest (n = 1). The estimated cumulative incidence of such major clinical events at 35 years of age was 8%—with lowest risk for TGA-IVS (5%) and highest for TB-DORV (23%).

What can be concluded from this large-scale nationwide registry? Firstly, patients with TGA who survive until ASO have a low mortality rate in the first decades after the surgical repair. The reported 30-day postoperative mortality of 0.9% in the registry may be underestimated compared to other real-world data, but other reports confirm that ASO can currently be performed at a low surgical risk. Secondly, the long-term outcomes depend on the type of TGA with best results for patients with TGA-IVS followed by TGA with ventricular septal defect and TB-DORV. Thirdly, a significant proportion of the patients will need subsequently re-intervention, with RVOT, LVOT, and arrhythmia been the most common causes (Figure 1). Fourthly, major clinical events are not uncommon during the follow-up.

**Figure 1:**
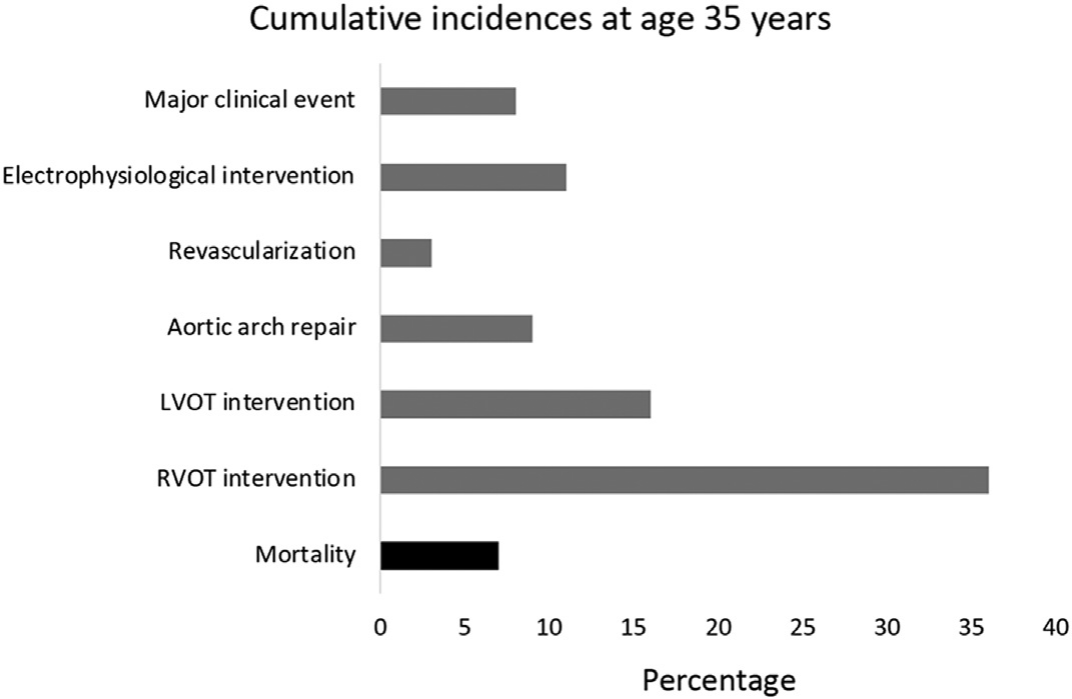
**Estimated Cumulative Incidences at Age 35 Years for Patients With Transposition of the Great Arteries Repaired With Arterial Switch Operation** LVOT = left ventricular outflow tract; RVOT = right ventricular outflow tract.

Despite surgical treatment of TGA has evolved to offer the patients a safe repair with ASO, the expectation that these patients have a normal circulation with low rates of anatomical sequelae and clinical events must be reconsidered. As described in guidelines, patients undergoing ASO should routinely be followed at centers with expertise in congenital heart disease. The visits must include assess for symptoms, clinical examination, echocardiography, and screening for arrhythmia for early detection of residual lesions what might need to be addressed. Furthermore, guidance in lifestyle including prevention of infectious endocarditis is paramount. Also, the surgical community needs to reflect on these findings and consider whether ASO procedure can be further refined to avoid RVOT and LVOT obstructions, preserve the neo-aortic valve function, and mitigate the risk of arrhythmias.

The journey for treatment of TGA has come a long way, but we must continue our efforts to improve the life for these patients.

## Funding support and author disclosures

The author has reported that they have no relationships relevant to the contents of this paper to disclose.
